# A Jordanian Multidisciplinary Consensus Statement on the Management of Dyslipidemia

**DOI:** 10.3390/jcm12134312

**Published:** 2023-06-27

**Authors:** Eyas Al Mousa, Sayer Al-Azzam, Mohammad Araydah, Reema Karasneh, Mohammad Ghnaimat, Hanna Al-Makhamreh, Abdelkarim Al Khawaldeh, Muneer Ali Abu Al-Samen, Jihad Haddad, Said Al Najjar, Hatem Alsalaheen Abbadi, Ayman J. Hammoudeh

**Affiliations:** 1 Jordanian Atherosclerosis and Hypertension Society, Amman 11942, Jordan; eyasalmousa@hotmail.com; 2 Clinical Pharmacy Department, Faculty of Pharmacy, Jordan University of Science and Technology, Irbid 22110, Jordan; salazzam@just.edu.jo; 3 Princess Basma Teaching Hospital, Irbid 22110, Jordan; mohaari98@gmail.com; 4 Department of Basic Medical Sciences, Faculty of Medicine, Yarmouk University, Irbid 21163, Jordan; reema.karasneh@yu.edu.jo; 5 Jordan Society of Internal Medicine, Amman 11942, Jordan; ghnaimat@hotmail.com; 6 Jordan Society of Nephrology, Amman 11942, Jordan; 7 Cardiology Division, School of Medicine, University of Jordan, Amman 11942, Jordan; hmakhamreh@hotmail.com; 8 The Jordanian Society of Endocrinology Diabetes & Metabolism, Amman 11942, Jordan; drabdkhawaldeh@gmail.com; 9 Jordanian General Practitioners Society, Amman 11942, Jordan; muneeral99@yahoo.com; 10 Scientific Committee, Jordanian Atherosclerosis and Hypertension Society, Amman 11942, Jordan; haddad_jihad@yahoo.gr; 11 Cardiology Department at Albasheer Hospital (MOH), Amman 11942, Jordan; sabdelrahman79@icloud.com; 12 Jordan Cardiac Society, Amman 111942, Jordan; habbadi03@yahoo.com; 13 Scientific Committee, Cardiovascular Academy Group of the Jordan Cardiac Society, Amman 11942, Jordan

**Keywords:** atherosclerotic cardiovascular diseases, dyslipidemias, cholesterol, triglycerides, low-density lipoproteins, high-density lipoproteins, apolipoprotein B, total cardiovascular risk, treatment, lifestyle, drugs

## Abstract

Atherosclerotic cardiovascular disease (ASCVD) is the primary contributor to global mortality rates, which significantly escalates healthcare expenditures. Risk factors for ASCVD (including dyslipidemia) frequently present in clusters rather than separately. Addressing these risk factors is crucial in the early initiation of a comprehensive management plan that involves both lifestyle modifications and pharmacotherapy to reduce the impact of ASCVD. A team of Jordanian professionals from various medical organizations and institutes took the initiative to create a set of guidelines for dyslipidemia screening and therapy. A detailed, comprehensive literature review was undertaken utilizing several databases and keywords. This consensus statement provides recommendations for dyslipidemia management in Jordanians on several issues including cardiovascular risk estimation, screening eligibility, risk categories, treatment goals, lifestyle changes, and statin and non-statin therapies. It is recommended that all Jordanian individuals aged 20 years old or older undergo lipid profile testing. This should be followed by determining the level of cardiovascular risk depending on the presence or absence of ASCVD and cardiovascular risk factors, eligibility for lipid-lowering therapy, and the target low-density cholesterol serum level to be achieved. In conclusion, prioritizing the management of dyslipidemia is of the utmost importance in improving public health and reducing the burden of cardiovascular diseases.

## 1. Introduction

The worldwide incidence of atherosclerotic cardiovascular disease (ASCVD) has witnessed a substantial rise, surging from approximately 271 million cases in 1990 to 523 million cases in 2019 [1]. Furthermore, ASCVD is the leading cause of global mortality, imposing a significant financial burden on healthcare systems [2,3]. In 2019, approximately 17.9 million deaths worldwide were attributed to ASCVD, which accounted for 32% of all fatalities, with heart attack and stroke deaths accounting for 85% of these fatalities [4].

The estimated prevalence of ASCVD in the Middle East was estimated to be 10.1% according to a recent systematic review and meta-analysis [5]. In Jordan, non-communicable diseases accounted for approximately 80% of all fatalities, with ASCVD emerging as the primary cause of these fatalities by accounting for approximately 37% of total deaths in 2018 [6,7].

The traditional risk factors for ASCVD (dyslipidemia, diabetes mellitus (DM), hypertension, smoking, family history of premature ASCVD, obesity, and sedentary lifestyle) often exist in clusters, rather than individually, thus increasing the risk of the development of atherosclerosis in a multiplicative rather than cumulative manner [8]. Therefore, the early detection of ASCVD and its risk factors is crucial in the early initiation of a comprehensive management plan that involves both lifestyle modifications and pharmacotherapy. In fact, by addressing behavioral risk factors such as tobacco use, unhealthy diet habits, obesity, lack of physical activity, and alcohol consumption, a majority of the atherosclerotic illnesses can be avoided [9,10].

The Jordanian population has been the subject of numerous studies examining the occurrence of risk factors associated with ASCVD [11,12,13,14]. In one study, a total of 1449 healthy participants were enrolled [11]. Dyslipidemia was the most common risk factor (74.6%), followed by elevated blood pressure (37.5%), obesity (32.5%), smoking (31.5%), and elevated random blood sugar (21.5%). Another study evaluated the prevalence of four ASCVD risk factors (hypertension, DM, smoking, and dyslipidemia) among Jordanians with coronary heart disease (CHD) [14]. The study found that a majority (95%) of the CHD patients had at least one of the four risk factors. In addition, it was found that the presence of DM, smoking, and elevated levels of low-density lipoprotein cholesterol (LDL-C) were notably more prevalent among individuals afflicted with CHD in comparison to their counterparts devoid of the condition [14].

Practicing physicians encounter two groups of individuals concerning ASCVD risk [15]. The first includes those with manifested diseases, such as CHD, stroke, and peripheral arterial disease. The second includes those who have one or more of the cardiovascular risk factors, with no clinically evident atherosclerosis. It is the second group of individuals that needs to have their cardiovascular risk estimated in order to prescribe pharmacological therapy according to the level of the cardiovascular risk. Cardiovascular risk is defined as the probability that an individual may experience an ASCVD event over a specific time period. The assessment of total cardiovascular risk is established by the cumulative impact of several risk factors [16]. Multiple scoring systems have been designed for cardiovascular risk estimation and incorporated in the majority of clinical practice guidelines for dyslipidemia management [17,18,19]. The cardiovascular risk assessment tools that have been used in the dyslipidemia guidelines were developed from cohorts in western societies that are very different from populations in other regions in the world, including the Middle East, thus limiting the generalizability of these risk estimation scores [20]. However, local experts in this region have developed risk score assessment tools that can be applied with confidence to tailor dyslipidemia therapy to each individual in the Middle East, including Jordan [21].

Dyslipidemia is defined as patterns of abnormal serum levels of total cholesterol (TC), LDL-C, high-density lipoprotein cholesterol (HDL-C), non-HDL-C, or triglycerides (TG) [22]. Dyslipidemia is a major risk factor for ASCVD and is related to poor dietary habits, tobacco use, a sedentary lifestyle, and genetic factors [22]. Moreover, abnormal LDL-C was found to have a fundamental role in the evolution of atherosclerosis and CHD and is the main target for every effective lipid-lowering strategy [23].

Multiple studies have investigated the occurrence and prevalence of dyslipidemia in both the general population and individuals diagnosed with ASCVD in Jordan [11,14,24,25,26,27]. In Jordan, the prevalence of dyslipidemia exceeds the global average, emphasizing the need for heightened awareness and proactive measures to address this significant public health concern. In cohort studies conducted between 2006 and 2022, dyslipidemia prevalence in the Jordanian population was extremely high and ranged from 75.7–81.6%, with a variable prevalence pattern of abnormally high serum levels of LDL-C reaching up to 75.9% [11,24,25,27]. On the contrary, a cross-sectional observational study examining the prevalence of TG and low LDL levels among 7641 European patients revealed that only 20.8% of the population were found to have dyslipidemia [28]. Additionally, Bilitou et al. conducted a study in the United Kingdom involving 279,211 patients that found that the prevalence of primary hypercholesterolemia and mixed dyslipidemia by 2019 did not exceed 23.5%, a significantly lower figure compared to the findings observed in Jordan [29]. It is worth mentioning that the LDL-C serum levels were not significantly different between Jordanian patients without CHD (132 ± 39.2 mg/dL) and patients with chronic CHD (135.4 ± 38.7 mg/dL) (*p* = 0.07) or compared to those with acute coronary syndrome (ACS) (130.7 ± 38.7 mg/dL) (*p* = 0.39) [30].

Several published clinical practice guidelines for the management of dyslipidemia from different medical societies have been generated based on epidemiological and clinical studies conducted in the western hemisphere [16,31,32,33,34,35]. These guidelines might not necessarily apply to other populations that have different baseline demographic features, risk factor prevalence, and statin eligibility. A local study demonstrated that more than 80% of statin-naïve patients with no DM who were admitted with acute myocardial infarction were found to be eligible for statin treatment compared to a rate of 49% of a similar population in the USA [36,37]. Regrettably, the country of Jordan faces a notable absence of refined clinical practice guidelines concerning the management and treatment of dyslipidemia, leaving healthcare professionals without established frameworks to navigate this highly complex area of medical practice. For these reasons, a uniform set of guidelines was developed by local experts to provide multidisciplinary and practical recommendations for healthcare professionals for the management of dyslipidemia in the Jordanian population. These guidelines will be reviewed on regular basis every several years to update the recommendations according to new published literature.

## 2. Materials and Methods

A panel of experts representing various medical societies and institutions in Jordan took the initiative to write a set of guidelines for the screening and management of dyslipidemia. The panel included cardiologists, endocrinologists/diabetologists, nephrologists, internists, general practitioners, nutritionists, and other health care specialists from the Jordan Atherosclerosis and Hypertension Society, the Jordan Cardiac Society, the Jordan Society of Nephrology, the Jordan Society of Internal Medicine, the Jordan General Practitioners Society, the Jordanian Ministry of Health, the University of Jordan, Jordan University of Science and Technology, Yarmouk University, and the Jordan Society of Endocrinology, Diabetes and Metabolism.

An extensive and comprehensive literature review using different databases, including PubMed, Web of Science, Medline, Scopus, and EMBASE, was conducted. The keywords that were used for the literature review were “dyslipidemia”, “hyperlipidemia”, “hypercholesterolemia”, “screening”, “risk category”, “cardiovascular risk”, “treatment”, “management”, “lipid-lowering therapy”, and “safety of pharmacotherapy”. Furthermore, the panel reviewed the latest recommendations from all available international guidelines. Another goal of the literature review was to identify studies that investigated ASCVD and lipid disorders in the Jordanian population. The search results were discussed in a number of meetings and a draft of the guidelines manuscript was discussed, edited and amended until unanimous agreement was reached based on the strength of the supporting evidence it provided and its applicability to Jordanian practice conditions.

## 3. Results and Discussion

The panel deliberated and reached consensus on five sequential measures to adopt in approaching Jordanian patients with dyslipidemia (Box 1). This set of measures would equip healthcare professionals with a comprehensive framework to methodically appraise and manage lipid disorders.

Box 1 **Steps to approach patients with dyslipidemia**

**Step 1:** Who needs to be screened?

**Step 2:** Measure the lipid profile.

**Step 3:** Estimate the cardiovascular risk level.

**Step 4:** Determine the target LDL-C to be reached.

**Step 5:** Prescribe a lipid-lowering management plan.

### 3.1. Step 1: Screening of Dyslipidemia

Age-specific dyslipidemia screening guidelines differ between countries; nevertheless, the decision to screen a Jordanian individual should always be established based on clinical reasoning and judgment. The panel recommended that lipid profile screening be performed for all Jordanian individuals 20 years of age or older, and, if the results are within normal limits, screening should be repeated every 5 years thereafter (Table 1). Individuals who are prescribed a lipid-lowering agent should have their blood tests repeated after 3 months to assess whether the LDL-C target has been reached. Moreover, all patients diagnosed with any of the diseases in Table 2 should be screened for dyslipidemia, regardless of their age. These screening rules were adopted and modified from the latest Canadian Cardiovascular Society Guidelines [32,38]. These conditions represent common risk factors for cardiovascular illnesses, as well as a range of inflammatory diseases that increase the chances of acquiring cardiovascular illnesses.

### 3.2. Step 2: Lipid Profile Measurement

Many clinical practices worldwide traditionally use fasting blood samples for lipid measurements. Recent comprehensive studies comparing fasting and non-fasting blood samples have found that the variations in most lipid measurements are minor [39,40,41]. In most studies, non-fasting blood samples have had higher levels of TG which were not that clinically significant for most individuals [39,42,43]. Based on this, the panel agreed that the use of either fasting (8–12 h) or non-fasting blood samples for the measurement of lipid parameters is reasonable. If a non-fasting sample shows a serum TG level of ≥400 mg/dL, then a fasting blood sample should be obtained.

Although LDL-C levels can be directly measured, the Friedewald formula is commonly used in many laboratories and studies to calculate LDL-C levels [31]. Calculated LDL-C levels have been found to underestimate LDL-C levels at concentrations of TG ≥ 200 mg/dL [44]. The calculation using the Friedewald formula is valid in cases where the TG concentration is below 400 mg/dL, and it becomes less precise when LDL-C levels are very low (below 50 mg/dL) [31]. Therefore, in patients with low LDL-C levels and/or hypertriglyceridemia (≤800 mg/dL), alternative formulas can be used or LDL-C levels can be directly measured [45].

Lp(a) testing is important in cardiovascular risk assessment since it helps to identify individuals who are at higher risk for cardiovascular events. Lp(a) testing may influence individualized treatment choices and help in the diagnosis of familial hypercholesterolemia [46]. However, it is worth mentioning that Lp(a) testing is not currently available in Jordan, which may explain its absence in our study. The scarcity of some diagnostic tests, such as Lp(a), may pose difficulties in particular locations or healthcare settings.

### 3.3. Step 3: Determine the Risk Category of the Individual

#### 3.3.1. Risk Factors for ASCVD

The risk factors for ASCVD (Table 2) were adopted from the 2020 American Association of Clinical Endocrinologists (AACE)/American College of Endocrinology (ACE) consensus statement on the management of dyslipidemia [47]. These cardiovascular risk factors were developed based on three major studies: the Framingham Heart study [48], the Multiple Risk Factor Intervention Trial (MRFIT) study [49], and the INTERHEART study [50], as well as using multiple previously published guidelines. These risk factors were classified into three groups: major risk factors, additional risk factors, and nontraditional risk factors. The major risk factors were advanced age, abnormal lipid profile, DM, hypertension, chronic kidney disease (CKD), smoking status, and ASCVD family history. Family history was defined as the occurrence of MI or sudden cardiac death in a female first-degree relative aged ≤65 years or in a male first-degree relative aged ≤55 years. The dyslipidemic triad was defined as the simultaneous presence of elevated TG, reduced HDL-C, and increased levels of low-dens LDL-C. In the Jordanian population, it was evident from multiple cohort studies that advanced age, hypertension, smoking status, poor lipid profile, obesity, DM, and others are major risk factors for developing ASCVDs [13,14,51,52].

Elevated levels of inflammatory markers, including high-sensitivity C-reactive protein (hsCRP) and lipoprotein-associated phospholipase (Lp-PLA2), are considered as nontraditional risk factors for ASCVD. Inflammation plays a substantial role throughout all phases of the complex pathological process known as atherosclerosis from the formation of fatty streaks through plaque progression and eventual complications such as plaque rupture or thrombosis [53].

According to the 2018 American College of Cardiology (ACC)/American Heart Association (AHA) for primary prevention guidelines, lipoprotein(a), often referred to as Lp(a), is considered an ASCVD risk enhancer when its values are ≥50 mg/dL or ≥125 nmol/L, especially in patients with family histories involving ASCVD [34]. Moreover, low HDL levels (male < 40 mg/dL and female < 50 mg/dL) are part of the metabolic syndrome which is a risk enhancer. Therefore, both low HDL and high Lp(a) levels are considered as risk markers that define patients who are at increased risk for future ASCVD, particularly when they are present in combination with other risk enhancers, and when treatment with statins is indicated.

**Table 2:** Risk factors for ASCVDs.

Major Risk Factors	Additional Risk Factors	Nontraditional Risk Factors
Advanced age	Obesity and abdominal obesity	↑ Lipoprotein
↑ TC	Family history of hyperlipidemia	↑ Clotting factors
↑ Non–HDL-C	↑ Small, dense LDL-C	↑ Inflammation markers (hsCRP and Lp-PLA2)
↑ LDL-C	↑ Apo B	↑ Homocysteine levels
Low HDL-C	↑ LDL particle concentration	Apo E4 isoform
DM	Fasting/postprandial hypertriglyceridemia	↑ Uric acid
Hypertension	PCOS	↑ TG-rich remnants
Chronic kidney disease	Dyslipidemia triad	
Smoker		
Family history of ASCVD		

**Adopted from the 2020 AACE/ACE consensus statement on the management of dyslipidemia** [46]. **ASCVD**, atherosclerotic cardiovascular disease; **Apo**, apolipoprotein; **DM**, diabetes mellitus; **HDL-C**, high-density lipoprotein cholesterol; **hsCRP**, high-sensitivity C-reactive protein; **LDL-C**, low-density lipoprotein cholesterol; **Lp-PLA2**, lipoprotein-associated phospholipase; **PCOS**, polycystic ovary **syndrome**; ↑, increased.

#### 3.3.2. Estimation of the Total Cardiovascular Risk in Apparently Healthy Individuals

The panel recommended adopting the Systematic Coronary Risk Evaluation 2 (SCORE2) and SCORE2-Older Person (SCORE2-OP) charts to calculate the overall 10-year risk of ASCVD events in apparently healthy individuals who do not have ASCVD, CKD, DM, or rare genetic lipid disorders [31]. Physicians who evaluate healthy Jordanian individuals for dyslipidemia are highly encouraged to use the risk charts designed for very high-risk regions (including Lebanon and Egypt) which have the closest demographic and risk factor prevalence to the Jordanian population (available at Heart Score (escardio.org)). These areas include eastern Europe and Arab countries similar to Jordan (e.g., Lebanon and Egypt).

The SCORE2 and SCORE2-OP charts were used previously to calculate cardiovascular risk for asymptomatic individuals based on age, sex, smoking status, total cholesterol level, and systolic blood pressure (SBP) measurement [54,55]. The previous risk estimation chart (SCORE) was used to calculate the 10-year risk for cardiovascular death, though it did not consider cardiovascular morbidity. In these updated charts, cardiovascular mortality and morbidity were taken into consideration, which better represents the overall burden of ASCVD. Moreover, the population was divided into three distinct groups (less than 50 years old, between 50 and 69 years old, and 70 years old or older). The SCORE2 chart should be used for individuals aged 40–69 years old, and the SCORE2-OP should be used for those aged 70 years or older.

#### 3.3.3. Cardiovascular Risk Categories

A crucial step in the management of dyslipidemia is risk categorization in order to set an LDL-C target serum level to be reached 8–12 weeks after initiation of lipid-lowering therapy. The panel recommended that all Jordanian individuals with lipid disorders should be categorized, according to the risk of ASCVD, into low, moderate, high, very high, or extremely high-risk categories based on the presence of ASCVD and other cardiometabolic diseases and risk factors (Table 3 and Table 4). These categories are intended for use in clinical settings and reflect the reality that preventative treatments are most beneficial for individuals who are at the greatest risk of a cardiovascular event. These risk levels were adopted from the European Society of Cardiology (ESC)/European Atherosclerosis Society (EAS) guidelines and the Polish guidelines [16,31,33].

### 3.4. Step 4: Determine the Target LDL-C Serum Level to Be Reached

Regardless of the local laboratory definitions of optimal lipid profile levels, the panel recommended the use of the optimal lipid profile levels presented in the ESC/EAS guidelines (Table 5) [16,31]. Given that a decline in LDL-C levels is accompanied by a similar decline in ASCVD occurrences [56], the consensus of the panel was to adopt the concept of LDL-C lowering as the main target for cardiovascular risk reduction and the use of lipid-lowering therapy. Even though achieving therapeutic LDL-C goals for the extremely high-risk group (<40 mg/dL) would appear to be quite challenging, it highlights the necessity for rigorous lipid-lowering medications to obtain LDL-C concentrations that are as low as possible, as quickly as possible, given the extreme safety of the commonly used lipid-lowering agents—statins, in particular—in the hundreds of thousands of individuals enrolled in clinical trials over the past three decades. Variances in therapeutic responses among individuals have been documented [57], and thus, the panel recommended that it is essential to customize treatment plans according to an individual’s needs.

### 3.5. Step 5: Management

#### 3.5.1. Lifestyle Modifications

Dietary variables can affect cardiovascular disease development through direct and indirect approaches by affecting conventional risk factors such as BP, plasma lipid concentrations, and blood sugar levels. Several epidemiological studies have found strong evidence that the incidence of cardiovascular events was reduced by increasing the consumption of vegetables (non-starchy), fruits, legumes, vegetable oil, nuts, whole grains, and yogurt and by reducing the intake of meats, refined carbohydrates, and salt [58,59]. The panel recommendations regarding lifestyle modifications in managing lipid disorders are presented in Table 6. These recommendations were adopted from the latest international guidelines [16,33,47].

Multiple clinical trials have documented the supplementary impact of dietary supplements, also known as nutraceuticals, on lipid-lowering medications. These supplements have demonstrated the potential to enable reductions in statin doses while maintaining the desired outcomes in terms of reduced TC and LDL-C levels [60]. Moreover, the use of these supplements has shown an ability to significantly mitigate adverse effects, a factor of considerable importance. Healthcare professionals should talk with patients about their lifestyle choices, encompassing dietary habits and the overall quality of their diet.

Since Jordanian individuals who are obese are at higher risk of type 2 DM and cardiovascular disease, the panel recommended that physicians should aim to reduce the BMIs of their patients to optimal levels [61,62]. Several studies have shown that increased physical exercise and activity among Jordanian individuals with CHD are associated with improved outcomes and quality of life, and thus, Jordanian physicians should encourage their patients to exercise more frequently [63,64,65]. Furthermore, it is recommended that Jordanian individuals with lipid disorders should reduce their consumption of trans-fats since it is evident that increased trans-fat intake is associated with a significantly increased risk of cardiovascular disease [66]. In one study, Jordanian individuals who were advised about dietary recommendations had a much higher likelihood of maintaining a healthy diet; thus, physicians should teach their patients about the importance of healthy lifestyles and eating habits [63]. In a cross-sectional, retrospective, multi-centered study conducted on Jordanian and Lebanese patients treated previously with statins, it was found that smoking, DM, and CHD were significant independent risk factors for not achieving optimal LDL-C level goals [67].

#### 3.5.2. Pharmacological Treatment

Alongside lifestyle modifications, pharmacotherapy is an essential component of lipid disorder management. The contemporary lipid-lowering pharmacological agents currently available in clinical practice in Jordan include statins, ezetimibe, proprotein convertase subtilisin/kexin type 9 (PCSK9) inhibitors (evolocumab), and small interfering ribonucleic acids (siRNAs) such as inclisiran, and these are used primarily to lower LDL-C levels.

##### Statins

Statins exert their therapeutic effect by reducing the liver synthesis of cholesterol through the competitive inhibition of the HMG-CoA reductase enzyme which is considered the rate-limiting step in the biosynthesis of cholesterol [68]. The resultant reduction in cholesterol intracellularly stimulates the higher expression of LDL-C receptors at the hepatocyte surface, thus increasing LDL-C uptake from the blood and resulting in a drop in plasma LDL-C levels.

Reductions in LDL-C concentrations in response to statin therapies are dose-dependent and differ among the various statins. The intensity of a statin is determined by the percentage of the LDL-C concentration reduction (Table 7).

The impacts of statins on improving lipid profile and lowering cardiovascular risk and mortality have been investigated and evidenced in multiple systematic reviews and meta-analyses [69,70,71,72,73,74,75,76]. For example, the use of high-intensity statins was associated with a nearly 55% reduction in serum LDL-C levels, and this reduction was associated with consistent and significant cardiovascular event reduction. Based on this, it has been recommended to use statins as first-line therapies to induce cholesterol reductions among Jordanian individuals, particularly moderate- and high-intensity statins [57,74]. Furthermore, based on latest ESC/EAS recommendations, it was recommended to titrate high-intensity statins up to the maximum-tolerated dose to reach treatment goals specific to each risk category [16].

Statins have demonstrated a significant capacity to effectively lower C-reactive protein (CRP) levels, functioning through a mechanism that is predominantly independent of LDL cholesterol levels [77]. In cases where there are elevated levels of inflammatory biomarkers (CRP), the administration of statin therapy leads to a more pronounced clinical benefit [78,79].

In individuals with several risk factors for ASCVDs or those with clinically stable ACS or ASCVD, even after aggressive high-intensity statin monotherapy, there is a substantial remaining risk for ASCVD [80,81,82]. Additionally, intolerance to statin or failure to achieve the required LDL-C level may restrict the use of high-dose statin monotherapy in some individuals and necessitate the addition of other non-statin lipid-lowering medications [83].

The panel emphasized the proven safety of statins which have very high benefit–risk ratios. Significant elevations in liver enzymes (>3 times the upper normal range) and CPK (>10 times the upper normal range), as well as the presence of myalgia are rare adverse event after statin therapy and they are resolved in a majority of cases after discontinuing the use of statins or reducing the dose. However, statin-associated muscle symptoms (SAMS) are still one of the most prevalent statin adverse events, especially in those on moderate- or high-intensity therapy. Furthermore, individuals who have autoimmune diseases and those who are on antiepileptic medications were found to have significantly higher rates of SAMS [84]. Therefore, healthcare providers must be aware of these adverse events and address them appropriately to ascertain the adherence of patients to their prescribed medications, thus reducing cardiovascular events.

Statin intolerance refers to the occurrence of one or more adverse events linked to statin treatment which improve or disappear when the dosage is reduced or when the medication is stopped. This condition can be categorized as either a complete inability to tolerate any statin dosage or partial intolerance where a patient is unable to tolerate the necessary dosage to achieve their specific treatment goal [85]. In order to categorize a patient as experiencing statin intolerance, it is necessary to have tried a minimum of two different statins, including at least one at the lowest recommended daily dose [85]. Symptoms of statin intolerance may be influenced by modifiable risk factors, and addressing these factors could potentially enhance statin tolerance in certain cases. These factors include hypothyroidism, alcohol use, vitamin D deficiency, strenuous exercise, obesity, DM, and alternative treatments that have the potential for drug–drug interactions [85]. In patients with established or suspected statin intolerance with high to extremely-high risk for ASCVD, non-statin therapies should be taken into consideration while continuing the efforts to identify a statin regimen that can be tolerated in order to prevent unnecessary delays in reducing atherogenic lipoproteins [85]. The panel recommended against discontinuing or reducing the doses of statins when LDL-C targets are achieved or very low serum levels are reached or in the absence of documented side effects.

##### Cholesterol Absorption Inhibitors (Ezetimibe)

Ezetimibe reduces the absorption of cholesterol (from both bile and diet) through the inhibition of brush border proteins in the small intestines, resulting in the activation of cholesterol conversion in the liver, thus reducing hepatocyte cholesterol levels and increasing the expression of LDL-C receptors. Ezetimibe reduces LDL-C levels by 15–25% [86,87,88].

The effect of ezetimibe in combination with statins on improving the morbidity, mortality, and rate of cardiovascular events has been illustrated in multiple RCTs in which LDL-C levels and cardiovascular events were significantly reduced by the addition of ezetimibe to statins compared to statin monotherapies in patients with ACS, CKD, DM, and familial hypercholesterolemia [89,90,91,92,93]. Based on this, ezetimibe should be added to ongoing statin therapies in Jordanian individuals whose LDL-C levels are not within the recommended levels. Moreover, ezetimibe should be used in individuals who are statin-intolerant.

##### PCSK9 Inhibitors

These medications act on the protein PCSK9, which is responsible for controlling LDL-C receptor expression at the surface of hepatocytes [94]. By the inhibition of these proteins, the number of expressed LDL-C receptors increases, thus promoting the uptake of LDL-C from the blood and consequently reducing blood LDL-C levels [95]. Evolocumab and alirocumab are PCSK9 inhibitors in clinical use worldwide. The former is available in Jordan. Both medications exhibit substantial reductions in the serum levels of LDL-C (up to 60%, based on the dose) [96,97]. Furthermore, reductions in TG concentrations (26%) and elevated HDL-C concentrations were observed with the use of these medications [98,99]. This was associated with significant reductions in cardiovascular events in patients on maximally tolerated statin doses and ezetimibe [100,101,102]. Thus, PCSK9 inhibitors should be used in Jordanian individuals who cannot tolerate statins and for those whose treatment goals are not achieved by statins and ezetimibe.

##### Inclisiran

Inclisiran is a recently approved lipid-lowering agent in Jordan. It is an siRNA molecule that targets the PCSK9 protein by inhibiting its biosynthesis. In a recent RCT (phase I and II), drops in serum LDL-C levels (50–55%) were achieved by twice-yearly subcutaneous injections of Inclisiran [103,104].

Two RCTs (the ORION-10 and ORION-11 trials) involved patients with ASCVD who had abnormally elevated LDL-C levels, despite being on maximum-tolerated statin doses, who were randomized to receive inclisiran or a placebo for a duration of 540 days [105]. Inclisiran significantly reduced LDL-C levels by 53.8% and 49.2% in the two studies, respectively. A recent pooled analysis found that inclisiran was associated with a 26% decline in composite major cardiovascular events (MACEs) (OR: 0.74, 95% CI: 0.58–0.94) [106].

The approved indications for Inclisiran are for adult patients with primary (non-familial or heterozygous familial) hypercholesterolemia or adults with mixed dyslipidemia in addition to lifestyle modifications in the two following conditions: (1) combined with statins or statins and other lipid-lowering agents in patients whose goal LDL-C levels are not achieved despite a maximum-tolerated dose of statin, and (2) alone or combined with other lipid-lowering agents in patients who exhibit intolerances to statins or those with contraindications for the use of statins.

##### Other Lipid-Lowering Agents

Several lipid-lowering agents exist that are either unavailable in the local market, not currently included in clinical trials, or primarily employed for purposes other than reducing LDL-C levels (Table 8).

##### Management of Hypertriglyceridemia

In individuals with severe hypertriglyceridemia (>500 mg/dL), it is recommended to use fibrate, omega-3 fatty acids, or niacin to reduce the risk of acute pancreatitis [47]. The primary objective is to lower triglyceride levels significantly, aiming to achieve a level well below 500 mg/dL [47]. For individuals with DM and presenting with triglyceride levels ranging from 200 to 499 mg/dl despite the satisfactory management of glycemic control, it is strongly advised to consider the use of fibrates as part of their therapeutic regimen [108].

##### Treatment Algorithm

**Figure 1:**
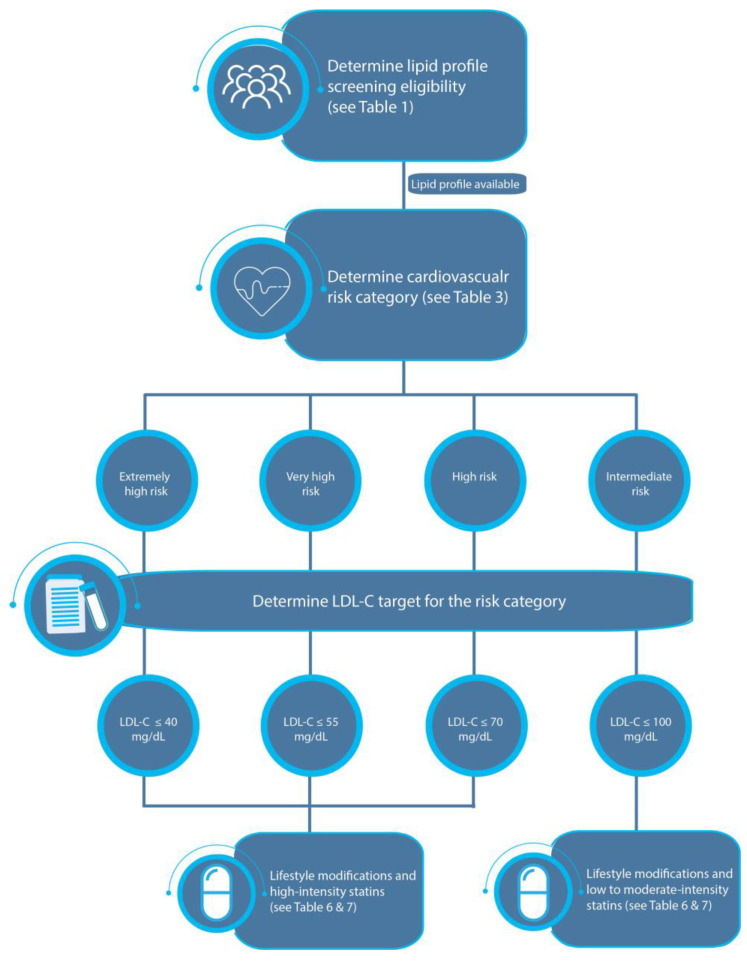
Management algorithm for an individual whose cardiovascular risk category is determined based on the presence of ASCVD, DM, CKD, and FH.

The panel decided to adopt the 2021 ESC dyslipidemia management algorithms and to simplify them for local healthcare practitioners in a four-step decision-making process [31]. Figure 1 describes lipid management in individuals whose cardiovascular risk is determined based on the presence of ASCVD, DM, CKD, and FH, with no need to use the SCORE risk calculator.

**Figure 2:**
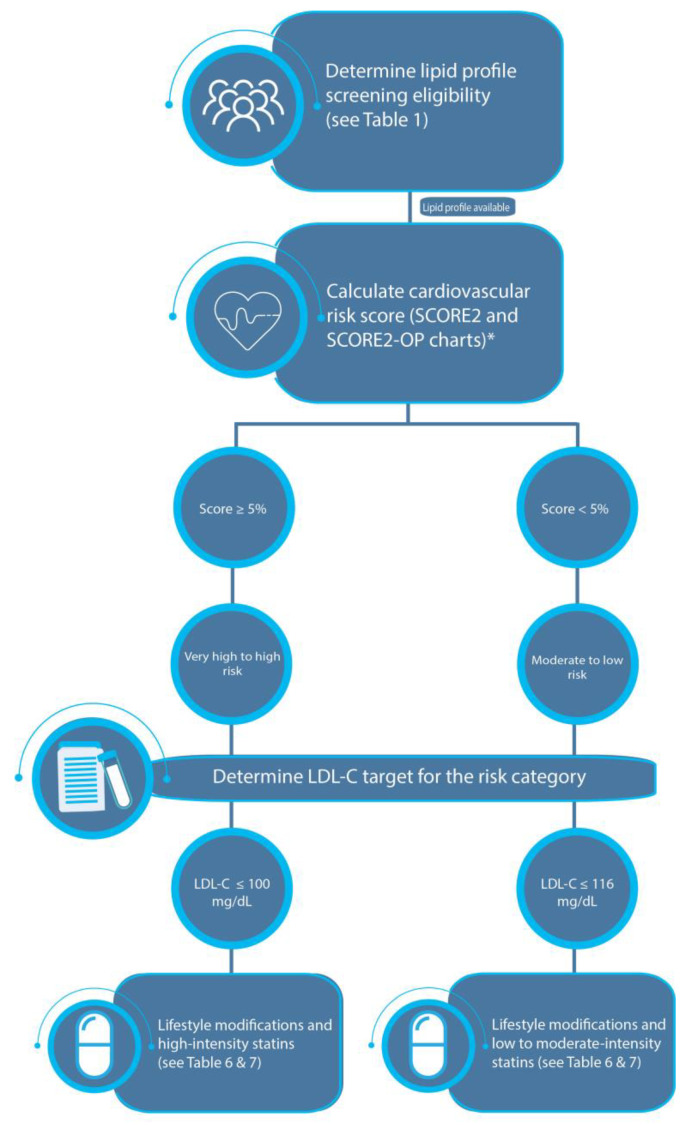
Management algorithm for individuals whose cardiovascular risk category is determined based on the SCORE2 and SCORE2-OP charts. *, available at https://heartscore.escardio.org/Calculate/quickcalculator.aspx?model=veryhigh&_gl=1*ry73gs*_ga*NzY5NDEyNTgzLjE2Nzg1NTI0MDc.*_ga_5Y189L6T14*MTY4MjE2MjM2Mi4zLjEuMTY4MjE2MjYzMS4wLjAuMA..&_ga=2.41447522.1773891670.1682162362-769412583.1678552407 (accessed on 20 April 2023). **SCORE2**, Systematic Coronary Risk Evaluation 2; **SCORE2-OP**, Systematic Coronary Risk Evaluation 2-Older Person.

Figure 2 describes lipid management in individuals with no clinically apparent ASCVD disease or other significant comorbid disease whose risk is determined based on the SCORE calculator. The original publication described nine levels of cardiovascular risk categories that were age-dependent (<50, 50–69, and ≥70 years). The panel recommended setting a score of 5% for all ages to separate moderate–low risk from higher risk individuals.

## 4. Conclusions

A team of Jordanian specialists have developed practical and patient-centered recommendations for the screening, categorization of ASCVD risk, and treatment of lipid disorders in the Jordanian population. These guidelines aim to reduce cardiovascular risk and are based on the extensive review of the available literature that is appropriate for the Jordanian population. The Jordanian specialists conducted an extensive literature review, examining relevant research and international guidelines. The main highlights of this study are the adoption of the SCORE2 and SCORE2-OP risk assessment charts, originally designed for high-risk regions in Europe, to assess cardiovascular risk in Jordan for those with no clinically overt ASCVD or associated morbidities. These risk charts were also applied to assess cardiovascular risk in other Arab countries, such as Lebanon and Egypt, that share similar characteristics with Jordan, including demographics and risk factor prevalence. Another notable aspect was the incorporation of an “extremely high-risk” category, which emphasizes the importance of the aggressive management of LDL-C levels (with a treatment goal of 40 mg/dL) for individuals in this category.

## 5. Top Ten Take-Home Messages

1. ASCVD is the leading cause of death in Jordan. Dyslipidemia is one of the major modifiable risk factors for ASCVD. Lowering LDL-C levels has been shown to decrease cardiovascular mortality and morbidity.
2. Screening for dyslipidemia is recommended for those who are 20 years of age and older. Screening at a lower age is indicated for certain high-risk individuals.
3. Screening for lipids can be performed in either a fasting or non-fasting state. However, if a non-fasting blood sample shows a TG level above 400 mg/dL, a fasting blood sample is indicated.
4. Lowering LDL-C levels to set target levels is the main objective in reducing cardiovascular risk.
5. Individual LDL-C serum level targets are determined by an individual’s level of cardiovascular risk (from extremely high risk to low risk).
6. The level of cardiovascular risk (from extremely high to low risk) is readily determined by the presence of clinical disease (ASCVD, DM, or chronic kidney disease, among other clinical diseases). In individuals who do not have any of these features, the level of risk (high risk, moderate risk, or low risk) should be estimated by calculating a 10-year risk score that utilizes certain clinical and laboratory features.
7. Lowering LDL-C levels should involve lifestyle modifications and lipid-lowering pharmacotherapy that includes statins and non-statins.
8. Statin therapy, particularly, high-intensity statin therapy, is indicated to achieve the target LDL-C level. Ezetimibe is added to statin therapy if the target LDL-C level is not achieved after 3 months of the maximally tolerated statin dose.
9. Two injectable lipid-lowering agents are available in Jordan: PCSK9i (Evolocumab), for SC administration using monthly or biweekly doses, and siRNA (inclisiran), administered subcutaneously twice yearly. Both medications are indicated for adult patients with primary hypercholesteremia or adults with mixed dyslipidemia.
10. Treatment for high TG levels is indicated when an individual’s levels are above 500 mg/dL, primarily to prevent pancreatitis. In diabetic patients with TG levels ranging from 200–499 mg/dL, despite adequate glycemic control treatment with fibrates, treatment is recommended.

## Figures and Tables

**Table 1:** Screening for dyslipidemia in Jordanian adults.

Men 20 years of age or older
Women 20 years of age or older
Regardless of age, all patients with any of the following conditions:
Clinical evidence of atherosclerosis
Abdominal aortic aneurysm
DM
Current smoker
Stigmata of dyslipidemia ^1^
Family history of premature cardiovascular disease ^2^
Family history of dyslipidemia
Chronic kidney disease ^3^
Obesity ^4^
Inflammatory diseases ^5^
HIV infection
Erectile dysfunction
Chronic obstructive pulmonary disease
History of hypertensive disorder of pregnancy

**Adopted from the Canadian guidelines** [32]. ^1^, corneal arcus, xanthelasma, and xanthoma; ^2^, men 55 years of age or younger and women 65 years of age or younger in first-degree relatives; ^3^, eGFR ≤ 60 mL/min/1.73 m^2^ or ACR ≥ 3 mg/mmol; ^4^, body mass index of ≥30; ^5^, for example, rheumatoid arthritis, vasculitis, psoriatic arthritis, and inflammatory bowel disease (modified by the panel); **DM**, diabetes mellitus.

**Table 3:** Cardiovascular risk categories considered for lipid-lowering therapy in the Jordanian population.

**Extremely high risk**	Individuals with a history of ACS who have any of the following conditions: Another vascular event that occurred in the preceding two years PVD or poly-vascular disease ^1^ Familial hypercholesterolemia DM and any of the following: lipoprotein (a) level of >50 mg/dL, high-sensitivity CRP level of >3 mg/dL, or CKD ^2^
**Very high risk**	Individuals who have any of the following conditions: Known ASCVD, including MI, unstable angina, stable angina, PVD, stroke, or TIA Coronary revascularization procedures, including PCI and CABG Imaging findings predictive of ASCVDs (coronary angiography, CT scan, or carotid ultrasound) DM with end-organ damage or three or more major cardiovascular risk factors Early-onset type 1 DM with a duration of more than 20 years Severe CKD ^3^ Family history of ASCVD or major cardiovascular risk factors
**High risk**	Individuals who have any of the following conditions: Markedly elevated TC (>310 mg/dL), LDL-C (>190 mg/dL), or BP (≥180/110 mmHg) Familial hypercholesterolemia without major cardiovascular risk factors DM without end-organ damage, with a disease duration of 10 years or more, and additional cardiovascular risk factors Moderate CKD ^4^
**Moderate risk**	Individuals who have any of the following conditions: DM (<35 years old for type 1 DM and <50 years old for type 2 DM) with a disease duration of less than 10 years
Low risk	Based on SCORE2 and SCORE2-OP results (see Table 4)

**Adopted from the ESC/EAS** [16] **and Polish** [33] **guidelines for the diagnosis and management of dyslipidemia**. ^1^, the presence of substantial atherosclerotic lesions in two or more of the three arterial beds (coronary arteries, cerebral arteries, and/or peripheral arteries); ^2^, estimated GFR of less than 60 mL/min/1.73 m^2^; ^3^, severe CKD (estimated GFR of less than 30 mL/min/1.73 m^2^); ^4^, moderate CKD (estimated GFR of between 30 and 59 mL/min/1.73 m^2^); **ACS**, acute coronary syndrome; **ASCVD**, atherosclerotic cardiovascular disease; **CABG**, coronary artery bypass graft; **CKD**, chronic kidney disease; **CRP**, C-reactive protein; **CT**, computer topography; **DM**, diabetes mellitus; **LDL-C**, low-density lipoprotein-cholesterol; **MI**, myocardial infarction; **PCI**, percutaneous coronary intervention; **PVD**, peripheral vascular disease; **TIA**, transient ischemic attack.

**Table 4:** Risk categories based on the SCORE2 and SCORE2-OP cardiovascular risk charts.

	Less Than 50 Years	Between 50 and 69 Years	70 Years or Older
**Low–moderate risk**	<2.5%	<5%	<7.5%
**High risk**	2.5%–<7.5%	5%–<10%	7.5%–<15%
**Very high risk**	≥7.5%	≥10%	≥15%

**Adopted from the 2021 European guidelines for the management of dyslipidemia [31]. SCORE2**: Systematic Coronary Risk Evaluation 2; **SCORE2-OP**: Systematic Coronary Risk Evaluation 2- Older Person.

**Table 5:** Treatment goals that should be achieved in the Jordanian population.

**TC**	Optimal < 200 mg/dL Borderline 201–240 mg/dL Elevated > 240 mg/dL
**TG**	Optimal < 150 mg/dL
**HDL-C**	Men > 40 mg/dL Women > 45 mg/dL
**LDL-C**	Extremely high risk: < 40 mg/dL Very high risk: < 55 mg/dL High risk: < 70 mg/dL Moderate risk: < 100 mg/dL Low risk: < 116 mg/dL

**HDL-C**, high-density lipoprotein-cholesterol; **LDL-C**, low-density lipoprotein-cholesterol; **TC**, total cholesterol; **TG**, triglycerides.

**Table 6:** Lifestyle modifications in the management of lipid disorders.

Decrease body weight and improve physical activity
Decrease intake of trans fats
Increase consumption of dietary fiber (fruit, vegetables, legumes, barley, and oats)
Maintain consumption of added sugars as less than 10% of the total intake
Avoid excessive alcohol intake
Smoking cessation
Rational dietary supplements such as monacolin, red yeast rice, phytosterols, dietary fiber, soy, policosanol, berberine, and n-3 fatty acids should be considered by a health professional

**Table 7:** Statin therapy based on intensity.

	High Intensity	Moderate Intensity	Low Intensity
Percentage of LDL-C reduction	≥50%	30–49%	<30%
Statin	Atorvastatin (40–80 mg) Rosuvastatin (20–40 mg)	Atorvastatin (10–20 mg) Rosuvastatin (5–10 mg) Simvastatin (20–40 mg) Pravastatin (40–80 mg) Fluvastatin XL (80 mg) Pitavastatin (1–4 mg)	Simvastatin (10 mg) Pravastatin (20 mg) Fluvastatin (20–40 mg)

**Adopted from the 2018 guideline for the blood cholesterol** [34].

**Table 8:** Other lipid lowering agents.

Medication	Effect	Status
**Fibrates**	Lowers TG by 25–50% and elevates HDL-C by 10–25%	Mainly used to prevent TG-induced pancreatitis
**Bile acids sequestrants (resins)**	Lowers LDL-C by 18–25% without substantial effects on HDL-C levels	Induces significant reductions in cardiovascular events in patients with dyslipidemia
**Omega 3 fatty acids**	Lowers TG levels by 45%	Mainly used to reduce high serum TG levels In patients at high risk with mild-to-moderate hypertriglyceridemia, the use of a high dose of Icosapent ethyl resulted in a notable decrease in ASCVD (REDUCE-IT)
**Bempedoic acid**	Lowers LDL-C levels by approximately 30% Combined with ezetimibe, may lower LDL-C levels by 50%	Treatment with Bempedoic acid significantly reduced LDL-C, non-HDL-C, TC, Apo B, and hs-CRP The use of Bempedoic acid reduced the risk of ASCVD in high-risk patients with statin intolerance (CLEAR-OUTCOMES)
**Obicetrapib**	Lowers non-HDL-C by approximately 44% and increases HDL levels by 165%	Phase 3 clinical trial underway (BROADWAY)

**Adopted from** [31,107]. **Apo B**, apolipoprotein B; HDL-C, high-density lipoprotein; **hs-CRP**, high-sensitivity C-reactive protein; **LDL-C**, low-density lipoprotein-cholesterol; **TG**, triglyceride.

## Data Availability

All relevant data are contained within the manuscript.
